# Pure CTCs, advanced WGS, and precise personalized combination therapies

**DOI:** 10.18632/oncoscience.362

**Published:** 2017-09-21

**Authors:** Brock A. Peters, John W. Park, Radoje Drmanac

**Affiliations:** Complete Genomics, Inc., San Jose, California; BGI-Shenzhen, Shenzhen, China

**Keywords:** CTC, WGS, LFR, ctDNA

In the past few years, use of liquid biopsy for next generation sequencing in cancer patients has expanded greatly, largely led by tests utilizing circulating tumor DNA (ctDNA). This is primarily because ctDNA is easy to isolate and appears to be released by many tumors [1]. However, analysis of ctDNA is not without its limitations: only in advanced cancers does it represent a significant percentage of the total cell-free DNA (cfDNA) in the blood, and the vast majority is found in small fragments of less than 200 base pairs. This has limited most assays utilizing ctDNA to mutation testing of less than 100 genes.

Circulating tumor cells (CTCs) have also found use in cancer diagnostics [2]. However, do to the complexity of isolating CTCs and technical challenges in generating high quality genomic data from a small number of cells, commercial development of CTC based assays has mostly focused around enumerating or counting of CTCs, which has been shown to have prognostic significance in a number of oncology settings. Our paper [3] is the first demonstration of a viable method for isolation and accurate whole genome sequencing (WGS) of CTCs. The process is highly automatable, and the cost is already within the range of current diagnostic tests. This was enabled by integrating novel CTC isolation techniques [4] with highly accurate long fragment read (LFR) WGS [5,6].

The first step of our process involved the highly pure isolation of CTCs. This is important because it meant we did not have to waste sequencing reads on normal cell DNA. In addition, our method allowed for accurate counting of mutations within each tumor cell. This enabled the detection of mutations found in just 10% of cells, but more importantly allowed for high confidence determination of mutations present in all of the CTCs. These results were confirmed by parallel sequencing of the primary tumor and metastasis. This suggests that CTCs can be used in lieu of multiple tissue biopsies to identify those mutations most likely to be found in all cancer cells in an individual patient, and as such ideal targets for therapy. As a strategy for robust liquid biopsy, analyzing CTCs as done in our study could be performed in lieu of tissue biopsy or surgery. In addition, using LFR DNA barcodes we were able to sequence and derive information from multiple CTCs without the need for separate isolation and sequencing of each individual CTC. For a clinical lab setting this is ideal as it allows for asimplified collection process and a single patient sample is contained in one tube. Importantly, the LFR process results in the independent barcoding of the complementary DNA strands. This is equivalent to doubling the number of CTCs per sample, a critical advantage because most clinical samples would be expected to have a small number of CTCs.

We detected somatic mutations in genes that could be targeted via multiple different drugs or therapeutic strategies, but would have been missed using current ctDNA assays. This comprehensive and accurate analysis of mutations allows for the identification of precise personalized therapies using appropriately targeted agents. In addition, WGS can in principle identify neoantigens and other non-driver mutations that can be used with novel treatment strategies, such as tumor-specific vaccines. Finally, we detected and phased all potential inherited cancer predisposing mutations and also phased somatic mutations affecting the same genes in order to determine if both alleles of a gene are inactivated. Inactivation of genes through inherited or inherited and somatic mutation can also point to specific therapies. Thus, comprehensiveness translates into opportunities for selecting the most effective personalized combination therapies.

As the technology continues to advance, we expect CTC based approaches to become increasingly important in cancer diagnostics and in precision cancer management. Improvements in CTC detection and isolation will allow more tumors to be detected at earlier stages of the disease. CTC isolation can be used for expression and proteomic analyses in conjunction with DNA sequence information for truly comprehensive molecular profiling. Advances in sequencing technology will continue to decrease the cost of the assay as well as provide more accurate and informative genome data. We continue to develop LFR to make it easy to automate, inexpensive, and informatically richer. In our current version [7] we have replaced wells in a 384-well format plate with beads in a single tube. We can now split the DNA content of cells across millions of compartments each with a unique barcode. This enables almost no overlap of long fragments within a compartment. The end result is sequence data that can be diploid de novo assembled with almost no errors [8]. Noninvasive, low cost, and high quality advanced LFR- WGS is ideally positioned for applications ranging from cancer screening to personalized cancer treatment to drug development.

In the field of cancer diagnostics, there will likely continue to be a place for both ctDNA and CTC based approaches. We believe that CTCs provide a unique source of high quality WGS data using advanced library and sequencing technologies like LFR and NGS reads >300 bases that will be critical to the goal of precision medicine.
